# Comparison of Clinical Outcomes of Myval vs. Acurate Neo Valves in TAVR: A Single-Center Experience

**DOI:** 10.3390/medicina62071366

**Published:** 2026-07-16

**Authors:** Murat Gökhan Yerlikaya, Ahmet Özderya, Ali Hakan Konuş, Mehmet Ali Maz, Fatih Gülçebi, Selim Kul, Ender Emre, Levent Korkmaz, Ali Rıza Akyüz

**Affiliations:** 1 Department of Cardiology, Trabzon Ahi Evren Thoracic and Cardiovascular Surgery Training and Research Hospital, University of Health Sciences, 61030 Trabzon, Turkey; 2 Department of Cardiology, Trabzon Kanuni Training and Research Hospital, 61250 Trabzon, Turkey; ahmetozderya@gmail.com (A.Ö.); fgulcelebi10@gmail.com (F.G.); 3 Department of Cardiology, Bingol State Hospital, 12000 Bingol, Turkey; alihakankonusede@gmail.com; 4 Department of Cardiology, Bayburt State Hospital, 69000 Bayburt, Turkey; malimaz29@hotmail.com; 5 Department of Cardiology, Kocaeli City Hospital, 41060 Kocaeli, Turkey

**Keywords:** Acurate Neo, balloon-expandable valve, Myval, self-expanding valve, transcatheter aortic valve replacement

## Abstract

*Background and Objectives*: Transcatheter aortic valve replacement (TAVR) has become an established treatment for severe symptomatic aortic stenosis, and continuous technological advances have led to the development of different transcatheter heart valve (THV) systems. This study aimed to compare the short- and mid-term clinical outcomes of the balloon-expandable Myval (Meril Life Sciences, India) and the self-expanding Acurate Neo (Boston Scientific, MA, USA) transcatheter heart valves during a mean follow-up of 18 months. *Materials and Methods*: This retrospective single-center study included consecutive patients who underwent transfemoral TAVR with either the Myval or Acurate Neo valve between January 2020 and September 2022. Demographic, clinical, laboratory, electrocardiographic, echocardiographic, procedural, and follow-up data were collected and compared. Clinical outcomes were evaluated according to the Valve Academic Research Consortium-3 (VARC-3) criteria. *Results*: A total of 147 patients were included (Myval, *n* = 116; Acurate Neo, *n* = 31). Predilatation was performed in all patients in the Acurate Neo group but in only 22 (18.9%) patients in the Myval group (*p* < 0.001). In-hospital mild paravalvular leak (PVL) was more frequent in the Acurate Neo group (38.7% vs. 20.6%; *p* = 0.038), whereas moderate or greater PVL did not differ between the groups. During a mean follow-up of 18 months (range, 10–32 months), mild PVL remained significantly more frequent in the Acurate Neo group (*p* = 0.028). No significant differences were observed between the two valve systems regarding mortality, permanent pacemaker implantation, or other major VARC-3 clinical outcomes. *Conclusions*: The Myval and Acurate Neo transcatheter heart valves demonstrated comparable short- and mid-term clinical outcomes in patients undergoing TAVR. Although mild PVL was more frequent with the Acurate Neo valve, mortality, permanent pacemaker implantation, and moderate or greater PVL were similar between the two valve systems, supporting the safety and effectiveness of both prostheses in the treatment of severe symptomatic aortic stenosis.

## 1. Introduction

Aortic stenosis (AS) is among the most common heart valve diseases in the world and in our country. The prognosis of advanced aortic stenosis causing cardiac symptoms has a high mortality rate. The mortality rate is 49% in the first 2 years and 57% in the first three years after the onset of cardiac symptoms [1]. Aortic balloon valvuloplasty and medical treatment do not reduce mortality in symptomatic advanced aortic stenosis [2].

Surgical aortic valve replacement (SAVR) is the oldest treatment method. As technology advances, transcatheter aortic valve replacement (TAVR) has become a preferred treatment method [3,4].

Despite continuous technological improvements and increasing operator experience, TAVR remains associated with several procedure-related complications, including paravalvular leak, conduction disturbances requiring permanent pacemaker implantation, vascular complications, stroke, acute kidney injury, and bleeding events. Although the incidence of these complications has declined with newer-generation transcatheter heart valves and refined implantation techniques, they continue to influence both short- and long-term clinical outcomes. Therefore, comparative studies evaluating the safety and performance of different transcatheter heart valve systems remain of considerable clinical importance.

Randomized controlled trials in high-risk patients have shown that TAVR, a new treatment modality, gives similar results to SAVR. Rapid gain of experience by operators has reduced complication and mortality rates [3]. In addition to high-risk patients, results have begun to be compared in medium–low risk patients [5,6]. There are two types of valves in terms of procedure, balloon expandable (BE) valve and self-expandable (SE) valve, and there are many studies in the literature comparing these valves from various aspects [7]. However, there are not many studies comparing Myval and Acurate Neo valves.

To our knowledge, comparative real-world data directly evaluating the balloon-expandable Myval and the self-expanding Acurate Neo transcatheter heart valves remain limited. Therefore, studies comparing the procedural characteristics, safety profile, and mid-term clinical outcomes of these two valve platforms may provide clinically relevant information to guide device selection in contemporary TAVR practice.

In this study, we aimed to evaluate the short-term and mid-term results of 2 different aortic valves (Myval, Acurate Neo) used in TAVR procedures in our center and not compared with each other before, and to contribute to the literature.

## 2. Method

### 2.1. Design of the Study

Our study is a retrospective single-center observational study. All studies that underwent TAVR with Myval (Meril Life Sciences Pvt. Ltd., Vapi, India) and Acurate Neo (Boston Scientific, Marlborough, MA, USA) valves between 1 January 2020 and 1 September 2022 were included. Valves that underwent TAVR, except Myval and Acurate Neo valves, were not included in the maintenance. Electrocardiography (ECG) data, echocardiography data, demographic characteristics, blood test data and clinical follow-ups of patients who woke up from the specified schedule before and after the procedure were accessed using the data system in our center. Products that could not be reached from various parts were excluded from the study.

The primary aim of our study was to examine periprocedural complications and survival between the two studied patient groups. The secondary aim was to compare clinical, echocardiographic, and procedural outcomes, including permanent pacemaker implantation, acute kidney injury, paravalvular aortic regurgitation, left ventricular ejection fraction, cerebrovascular events, and cardiac functional capacity. Additionally, follow-up outcomes at 1 month and a mean follow-up of 18 months were evaluated according to the VARC-3 criteria.

The study design was approved by the local ethics committee in accordance with good clinical practice and was conducted in accordance with the Declaration of Helsinki. Ethics committee approval date: 26 October 2022, Number: 10496660-11.

### 2.2. Obtaining Data

Between 1 January 2020 and 1 September 2022, 147 patients with Myval (Meril Life Sciences Pvt. Ltd., Vapi, India) and Accurate Neo (Boston Scientific, Marlborough, MA, USA) heart valves who met the exclusion and inclusion criteria were contacted at our center. Baseline, demographic and periprocedural data of all patients were obtained. Laboratory data of the patients included renal and liver function tests, complete blood count, electrolytes, fasting blood sugar, high-sensitivity Troponin I (hs-Troponin I), C-reactive protein (CRP) and lipid panel from fasting peripheral venous blood samples taken in the morning before the TAVR procedure. Glomerular filtration rate of the patients was calculated using the MDRD formula.

All patients underwent electrocardiography before and after the TAVR procedure.

Patients who underwent echocardiography were first placed in the left lateral decubitus position. Echocardiographic examination was performed using a 3.5 MHz transducer and Philips EpiQ-7 system (X5 probe, Philips^®^ Medical Systems, Andover, MA, USA). Optimal apical images were opened and the ejection fraction of the left ventricle was calculated by the Simpson method using software modifications in the echocardiography device. These are the velocity, velocity time integral (VTI), mean and maximum gradient (min-max gradient) data in LVOT and the aortic valve. The average of the measurements taken in 3 beats in sinus rhythm and in 5 beats in alternating rhythms such as atrial fibrillation was taken.

**Figure 1:**
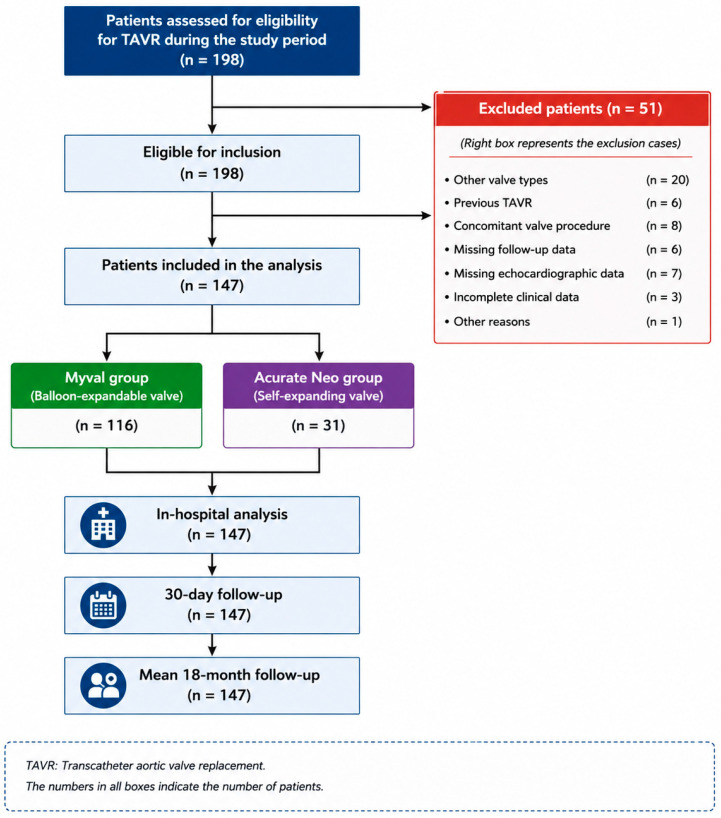
Study flow diagram. Flowchart illustrating patient selection, exclusion criteria, study group allocation, and clinical follow-up. Patients who underwent transcatheter aortic valve replacement (TAVR) with either the balloon-expandable Myval valve or the self-expanding Acurate Neo valve between January 2020 and September 2022 were screened for eligibility. Patients receiving other transcatheter heart valve systems or those with incomplete clinical or follow-up data were excluded. The final study population consisted of 116 patients treated with the Myval valve and 31 patients treated with the Acurate Neo valve. All included patients underwent in-hospital evaluation, 30-day follow-up, and a mean follow-up of 18 months.

In-hospital, 1-month and mean 18-month all-cause mortality, vascular complications, cerebrovascular events, acute renal failure, device success, bleeding complications and other events were defined according to the VARC-3 report [8]. Causes of death of patients were classified into 2 groups: cardiac and non-cardiac. Deaths of unknown cause were included in the cardiac group. The registration flow chart is shown in Figure 1.

### 2.3. TAVR Procedure

All TAVRs were implanted by the same team. Heart valve selection, size, and placement were determined by the implantation team. On the recommendation of anesthesia, almost all of the patients were sedated and analgesia was administered in such a way that they did not lose consciousness. Heparin was administered intravenously throughout the procedure and the activated clotting time was kept at >250 s. Activated clotting time was measured at half-hour intervals. In patients without peripheral vascular anomalies or pathology, the right femoral artery was used for cardiac implantation, the left femoral artery was used for aortic valve evaluation with contrast medium, and the left femoral vein was used for temporary pacemaker implantation. The contrast medium was standardized and at least 30 mL was administered at a rate of 16 m/s.

Valve selection was performed by the institutional Heart Team after comprehensive evaluation of each patient. The choice between the Myval and Acurate Neo transcatheter heart valves was based on multiple clinical and anatomical factors, including annular dimensions, vascular access characteristics, calcification distribution, device availability, and the operator’s judgment. Therefore, valve allocation was not randomized but reflected routine real-world clinical decision-making.

Immediately before implantation, a temporary pacemaker was used to increase the heart rate to 180 beats per minute. Implantation was performed in 3–5 s according to the characteristics of the patients. Implantation success was defined according to VARC-3 criteria.

Percutaneous closure devices for hemostasis at the end of the TAVR procedure were recorded: ProGlide 6-Fr, Perclose or Prostar XL Suture devices. Surgical cessation of hemostasis in case of failure to achieve hemostasis.

After a successful TAVR procedure, patients were given single antiplatelet therapy. Patients with atrial fibrillation (AF) who underwent coronary stenting procedures were given individualized antiplatelet and anticoagulant therapy considering the risk of bleeding and stent thrombosis. After the TAVR procedure, paravalvular leak (PVL) and valve gradient were evaluated echocardiographically and angiographically.

### 2.4. Follow-Up Procedure

Patients’ hospitalization, 1st-month and average 18-month follow-up data were accessed using our hospital data system. After the echocardiography procedure, the patient was evaluated with transthoracic echocardiography at 1st month and averaged 18 months. The evaluation was performed according to the most up-to-date guidelines and transthoracic echocardiography data were recorded [9].

### 2.5. Statistical Method

Statistical analyses were performed using IBM SPSS Statistics for Windows, Version 20.0 (IBM Corp., Armonk, NY, USA). Categorical variables are presented as frequencies and percentages and were compared using the Pearson chi-square test or Fisher’s exact test, as appropriate. The distribution of continuous variables was assessed using the Kolmogorov–Smirnov test, and homogeneity of variances was evaluated before selecting the appropriate statistical test. Normally distributed continuous variables are presented as mean ± standard deviation (SD) and were compared using the independent-samples *t* test. Non-normally distributed continuous variables are presented as median (minimum–maximum) and were compared using the Mann–Whitney *U* test. All statistical tests were two-sided, and a *p* value < 0.05 was considered statistically significant.

## 3. Results

A total of 147 patients were included in our study. In-hospital, 1-month and mean 18-month follow-ups were performed. 116 patients received Myval THV (70 females and 46 males; mean age 79.91 ± 7.44 years) and 31 patients received Acurate Neo valve implant (23 females and 8 males; mean age 81.65 ± 6.49 years). Demographic characteristics, functional capacities and medications of the patients are given in Table 1. Pre-procedure laboratory parameters, electrocardiography and echocardiography parameters are given in Table 2.

All of our patients underwent the procedure using a transfemoral approach and conscious sedation. While predilation was applied to all patients in the Acurate Neo valve group, 22 patients (18%) in the Myval THV group underwent predilation (*p* < 0.001). Postdilation was required in 5 (16%) patients in the Acurate Neo group, while postdilation was required in 6 (5%) patients in the Myval group (Table 3 *p* = 0.039). No statistical difference was found in peripheral closure and other procedure parameters. The median post-procedural length of hospital stay was 3 days (range, 1–37) in the Myval group and 3 days (range, 1–10) in the Acurate Neo group, with no statistically significant difference between the groups (*p* = 0.207). During the procedure, a 26 mm Myval valve balloon was removed from one patient in the Myval group and implanted into the descending aorta. No patient required general anesthesia or emergency surgery. No cerebral protection device was placed in any patient during the procedure.

No death was detected in any patient during the procedure. According to VARC-3 criteria, periprocedural death occurred in 5 (4.3%) patients in the Myval THV implanted group and 2 (6.4%) in the Acurate Neo group. The causes of mortality due to myval THV include MI due to LMCA obstruction, cardiac tamponade and pulmonary edema due to right ventricular lead, major vascular bleeding (Type 4), left ventricular obstruction (LVO) and cardiogenic shock. The causes of mortality due to Acurate Neo THV are aortic dissection and pulmonary edema (Table 4).

Although valve embolism [1 (0.9%)], suicide ventricle [1 (0.9%)], coronary artery obstruction [1 (0.9%)], periprocedural MI [1 (0.9%)] and cardiac tamponade [4 (3.4%)] were rarely detected in the Myval valve group, no statistical difference was observed between the two groups. When PVL was analyzed according to VARC-3 criteria, PVL was detected in 85 patients (72%) in the Myval THV group and 17 patients (54%) in the Acurate Neo group (*p* = 0.048). Mild PVL was detected in 24 patients (20.6%) in the Myval THV group and in 12 patients (38.7%) in the Acurate Neo group (*p* = 0.038). A statistically significant difference was found between the parameters with no PVL and those with mild PVL.

A comparison of the patients’ 30-day follow-up data after the procedure was made (Table 5). No difference was found between the two groups in terms of VARC-3 criteria in terms of device success (*p* = 0.804), early safety (*p* = 0.628), mortality (*p* = 0.806), hospitalization (*p* = 0.801), and other clinical and echocardiographic follow-up data. One-month mortality occurred in 9 patients (7.7%) in the Myval THV implanted group and 2 patients (6.4%) in the Acurate Neo group. Severe acute respiratory syndrome coronavirus was added to the list of Myval THV-related mortality causes, while the number and causes of Accurate Neo THV-related mortality did not change.

The patients’ mean 18-month (minimum 10 months, maximum 32 months) follow-up data were compared. Mortality during the mean 18-month follow-up occurred in 25 patients (21.5%) in the Myval THV group and 3 patients (9.6%) in the Acurate Neo group (Table 6). Although mortality was numerically higher in the Myval group, this difference did not reach statistical significance. Clinical efficacy according to the VARC-3 criteria was comparable between the two groups [74 (63.8%) vs. 20 (64.5%); *p* = 0.941]. Mild PVL remained significantly more frequent in the Acurate Neo group [12 (38.7%) vs. 23 (19.8%); *p* = 0.028], whereas no statistically significant differences were observed in moderate PVL, functional capacity, neurological events, permanent pacemaker implantation, or other follow-up outcomes.

## 4. Discussion

In this retrospective single-center study, we compared the short- and mid-term clinical outcomes of the balloon-expandable Myval and the self-expanding Acurate Neo transcatheter heart valves. The principal findings of our study were that both valve systems demonstrated comparable procedural success, mortality, and most clinical outcomes during follow-up. However, mild paravalvular leak was significantly more frequent in the Acurate Neo group, whereas no significant differences were observed in moderate or greater PVL, permanent pacemaker implantation, or mortality. These findings are discussed below in the context of the current literature.

Technical success, periprocedural mortality, device success, early safety, cerebrovascular events, and mortality differences are the primary parameters compared in studies comparing BE and SE valves. Barki et al. compared the Myval and Evolute R valve systems and found no statistically significant difference in periprocedural mortality, technical success, cerebrovascular events, and all-cause mortality. However, unlike our study, Barki et al. found a difference in device success and early safety rates [10]. We believe that this is due to the statistical difference between moderate and severe PVL formation in the study by Barki et al.

Patients who undergo TAVR may require a permanent pacemaker due to various mechanisms [11]. In the study conducted by Than JLM et al., it was shown that permanent pacemaker rates were higher than those in SE valve systems [12]. In the study conducted by Barki et al., BE and SE valve systems were compared; no statistical difference was found in permanent pacemaker rates [10]. In our study, no statistical difference was found between the two groups in terms of permanent pacemaker. We think that the reason for this is that although the implantation site was determined as 70–30% according to the landing site as a standard in our study, higher implantation was performed in selected cases, especially in the presence of RBBB in the basal ECG.

Previous studies comparing BE and SE valve systems have investigated procedural and mortality differences. PVL also has an important place among the causes of mortality [13]. PVL is a common complication and cause of mortality in patients undergoing TAVR. There are studies showing that moderate and severe PVL increases mortality. There are also studies showing the negative effect of mild PVL on mortality [14]. Studies have shown that the incidence range of PVL is wide. According to previous studies, the incidence of mild to severe PVL in patients undergoing TAVR ranges between 0.6% and 40% in BE valve systems and between 2% and 22% in SE valve systems [15,16]. In our study, no difference was found between the two groups compared in terms of moderate and severe PVL rates. There are studies showing that the frequency of moderate and severe PVL increases with low body mass index and high initial aortic valve mean gradient [17]. In our study, the lack of difference between the groups in moderate and severe PVL may be explained by the lack of difference between body mass index and baseline aortic valve mean gradient.

Aortic valve calcification is directly associated with mortality in patients undergoing TAVR. Aortic valve calcification is also an important risk factor for PVL. Studies have found that increased aortic valve calcification increases the likelihood of developing PVL. Even Viktor Mauri et al. investigated the effect of localized calcification on PVL in patients undergoing TAVR and recommended the use of the BE valve to reduce the development of PVL if the amount of calcification was high. In the same study, they emphasized that the amount of calcification as well as whether the calcification is symmetric or asymmetric has an effect on PVL monitoring. They showed that self-expanding THV may increase the formation of PVL in patients with asymmetric calcification [18]. Luigi FM Di Artino et al. showed that the Agatston score was a predictor in their study investigating the predictive parameters of PVL after TAVR [19]. Although there was no statistically significant difference between multi-slice computer tomography and aortic valve calcifications (Agatston Score) in our study, numerically, the amount of calcifications was higher in the Myval THV group. However, while there was no statistically significant difference between the two groups in the rates of moderate and higher PVL, mild PVL was significantly higher in the Acurate Neo group than in the Myval group. We think that the higher incidence of mild PVL in the Acurate Neo group may be due to the distribution of calcification and procedural features.

Recent studies have shown that mild PVL also increases mortality [15,20]. In our study, a statistically significant difference was found in the incidence of mild PVL between the two groups of in-hospital and mean follow-up of 18 months. However, in our study, there was no significant difference between the mortality data in the in-hospital and 18-month mean follow-up after the procedure. Although the mortality rate at the mean 18-month follow-up was numerically higher in the Myval group than in the Acurate Neo group, this difference did not reach statistical significance. Therefore, the observed numerical difference should be interpreted cautiously and does not support a statistically significant difference in long-term mortality between the two valve systems.

There are conflicting studies regarding the effect of predilation on PVL. However, none of these studies found a difference in aortic regurgitation [21]. Operators may choose not to perform predilation to avoid complications that may occur due to prolonged procedure time. However, as in a case performed in our center, the valve may strip off the balloon and cause undesirable results [22]. Based on our experience in our first series of 25 cases [23]. Where Myval THV was applied in our center, in cases where the valve moves backwards on the balloon, we completely remove the valve and re-tighten it. Then, we routinely dilate the sheath with an 18 F dilator before the procedure. In our study, a statistically significant difference was found between the two groups in terms of the use of the pre-dilatation method. Direct implantation was preferred in the BE group whenever possible. Deharo P. et al. showed that TAVRs directly implanted without pre-dilatation yielded successful results and that the procedure could be simplified [24]. The DIRECT study published in 2019 showed that there was no significant difference in device success between the THV implanted without predilation and the THV system implanted with predilation [25]. Hemodynamic disorders such as severe aortic insufficiency may occur after predilation, which may cause pulmonary edema, as we encountered in 1 patient in our study. Similar results are available in the literature [26]. Another possible negative consequence of predilatation is conduction disturbances. In our study, no statistical difference was found in conduction disturbances before and after the procedure. However, it was noted that the LBBB that developed after the procedure was numerically higher, although not statistically significant. It is also known that many procedural parameters may be effective in the formation of conduction disturbances and predilatation alone may not be responsible [27].

An additional consideration when interpreting our findings is the marked difference in predilatation strategy between the two valve groups. In our cohort, predilatation was performed in all patients receiving the Acurate Neo valve but in only a minority of patients treated with the Myval valve. This difference reflects both operator preference and the distinct implantation characteristics of the two transcatheter heart valve systems in routine clinical practice. Consequently, the observed difference in mild PVL should not be attributed solely to valve design, as procedural strategy may also have contributed to this finding. Likewise, conduction disturbances following TAVR are known to be multifactorial and may be influenced by implantation technique in addition to prosthesis-specific characteristics. Therefore, our results should be interpreted with consideration of these procedural differences.

Compared to surgical closure, closure with percutaneous devices reduces hospital stay and procedure times. In our study, Prostar was used in 6 patients in the Myval group but not in the Acurate Neo group and no statistical difference was found between the two groups. In our study, Proglide was mostly preferred because it is more convenient and practical. There was no statistical difference between the two groups in terms of Proglide use. Single proglide use was used in 45 patients in the Myval group but not in the Acurate Neo group. Dual Proglide use was applied to 52 patients in the Myval group and 30 patients in the Acurate Neo group. No statistical difference was observed between the two groups in terms of surgical closure method use. When vascular complications were compared, no statistical difference was found between the two groups. We think that this is due to the use of dual proglide in patients who are likely to develop vascular complications.

An additional finding of our study was the comparable rate of cardiovascular rehospitalization between the Myval and Acurate Neo groups during follow-up. Cardiovascular rehospitalization after TAVR is a multifactorial outcome and is frequently influenced by advanced age, frailty, progression of underlying heart failure, atrial fibrillation, coronary artery disease, renal dysfunction, and other patient-related comorbidities rather than prosthesis type alone [28,29,30]. Although cardiovascular rehospitalization was prospectively recorded as a predefined clinical outcome, the specific causes of rehospitalization were not systematically categorized in our retrospective database. Therefore, a detailed analysis according to the exact reason for rehospitalization could not be performed. This may explain the absence of a significant association between valve type and cardiovascular rehospitalization in our cohort.

Although the overall rates of bleeding and blood transfusion appeared relatively high in both groups, these findings should be interpreted in the context of the VARC-3 bleeding definitions, which include both minor and major bleeding events [8]. In addition, our study population consisted predominantly of elderly patients with severe symptomatic aortic stenosis, multiple comorbidities, and frequent use of antithrombotic therapy, all of which are well-recognized contributors to bleeding complications following TAVR [31,32,33]. Therefore, the observed bleeding rates likely reflect the baseline clinical characteristics of this high-risk population rather than differences related to the valve type itself.

### Our Limitations

This study has several limitations. First, it was a retrospective, single-center observational study and is therefore subject to the inherent limitations of retrospective analyses, including potential selection bias and residual confounding. Second, the study groups were unequal in size, with substantially more patients in the Myval group than in the Acurate Neo group. This imbalance reflects real-world clinical practice and may have influenced the statistical comparisons despite the absence of significant baseline differences between the groups. Third, transcatheter heart valve selection was not randomized but was based on operator discretion, anatomical characteristics, and device availability, which may have introduced additional selection bias. Fourth, the duration of follow-up varied among patients (10–32 months), precluding uniform time-to-event analyses such as Kaplan–Meier survival analysis. Furthermore, because of the relatively limited sample size, the imbalance between the study groups, and the low number of clinical events, propensity score matching or multivariable adjustment was considered but was not performed, as these approaches could have resulted in unstable statistical estimates and reduced analytical reliability. Finally, although cardiovascular rehospitalization was recorded as a predefined clinical outcome, the specific causes of rehospitalization were not systematically categorized in the retrospective database, preventing a more detailed analysis of rehospitalization events.

Therefore, larger prospective multicenter studies with balanced study groups, longer and standardized follow-up, and comprehensive event adjudication are warranted to validate the present findings.

## 5. Conclusions

In this retrospective single-center study, the balloon-expandable Myval and the self-expanding Acurate Neo transcatheter heart valves demonstrated comparable short- and mid-term clinical outcomes, with no significant differences in mortality, major adverse clinical events, permanent pacemaker implantation, or moderate or greater paravalvular leak during a mean follow-up of 18 months. Mild paravalvular leak was observed more frequently in the Acurate Neo group; however, this finding should be interpreted in the context of the markedly different predilatation strategies and procedural characteristics between the two valve systems. Overall, both prostheses provided favorable procedural and mid-term clinical performance, supporting their use as effective treatment options for patients with severe symptomatic aortic stenosis. Larger prospective randomized studies are warranted to further clarify the comparative long-term safety and efficacy of these valve systems.

## Figures and Tables

**Table 1:** Baseline demographic characteristics, comorbidities, and medications of the study population.

Variable	Myval (n = 116)	Acurate Neo (n = 31)	*p*-Value
**Baseline characteristics**			
Age, years	81 (76–86)	82 (77.5–87.5)	0.238
Female/Male, n	70/46	23/8	0.155
Body mass index, kg/m^2^	28.0 (25.0–31.0)	28.15 (24.0–31.0)	0.766
Hypertension, n (%)	93 (80.2)	28 (90.3)	0.188
Diabetes mellitus, n (%)	30 (25.8)	5 (16.1)	0.258
Current smoker, n (%)	25 (21.5)	9 (29.0)	0.380
Hyperlipidemia, n (%)	69 (59.5)	23 (74.2)	0.133
Chronic obstructive pulmonary disease, n (%)	26 (22.4)	6 (19.4)	0.714
Previous cerebrovascular event, n (%)	11 (9.5)	4 (12.9)	0.576
Coronary artery disease, n (%)	57 (49.1)	14 (45.2)	0.694
Previous myocardial infarction, n (%)	31 (26.7)	11 (35.5)	0.338
Previous percutaneous coronary intervention, n (%)	23 (19.8)	9 (29.0)	0.270
Coronary artery bypass grafting, n (%)	15 (12.9)	4 (12.9)	0.997
Atrial fibrillation, n (%)	38 (32.7)	9 (29.0)	0.146
**Medications**			
Loop diuretic, n (%)	80 (69.0)	23 (74.2)	0.770
Thiazide-like diuretic, n (%)	18 (15.5)	1 (3.2)	0.192
ACE inhibitor, n (%)	79 (68.1)	23 (74.2)	0.513
Angiotensin receptor blocker, n (%)	13 (11.2)	2 (6.5)	0.698
Beta-blocker, n (%)	94 (81.0)	27 (87.1)	0.432
Clopidogrel, n (%)	41 (35.3)	12 (38.7)	0.729
Aspirin, n (%)	55 (47.4)	18 (58.1)	0.292
Calcium channel blocker, n (%)	21 (18.1)	9 (29.0)	0.180
Statin, n (%)	65 (56.0)	15 (48.4)	0.599
Insulin, n (%)	9 (7.8)	0 (0.0)	0.109
Oral antidiabetic drugs, n (%)	25 (21.5)	3 (9.7)	0.135
Non-vitamin K antagonist oral anticoagulant, n (%)	40 (34.5)	11 (35.5)	0.161
Vitamin K antagonist, n (%)	7 (6.0)	0 (0.0)	0.161

Continuous variables are presented as median (interquartile range [IQR]). Categorical variables are presented as n (%). Continuous variables were compared using the Mann–Whitney *U* test, whereas categorical variables were compared using the chi-square test or Fisher’s exact test, as appropriate. Abbreviations: ACE, angiotensin-converting enzyme; AF, atrial fibrillation; ARB, angiotensin receptor blocker; ASA, acetylsalicylic acid; BB, beta-blocker; BMI, body mass index; CABG, coronary artery bypass grafting; CAD, coronary artery disease; CCB, calcium channel blocker; COPD, chronic obstructive pulmonary disease; CVE, cerebrovascular event; DM, diabetes mellitus; HL, hyperlipidemia; HT, hypertension; IQR, interquartile range; MI, myocardial infarction; NOAC, non-vitamin K antagonist oral anticoagulant; OAD, oral antidiabetic drug; PCI, percutaneous coronary intervention; VKA, vitamin K antagonist.

**Table 2:** Baseline laboratory, electrocardiographic, and echocardiographic characteristics of the study population.

Variable	Myval (n = 116)	Acurate Neo (n = 31)	*p*-Value
**Laboratory parameters**			
Hemoglobin, g/dL	11.65 ± 1.92	11.20 ± 1.93	0.259
Hematocrit, %	35.89 ± 5.51	34.45 ± 5.94	0.205
White blood cell count, ×10^3^/µL	7.51 (1.30–45.00)	8.10 (4.50–10.70)	0.803
Lymphocyte count, ×10^3^/µL	1600 (350–5700)	1630 (690–4270)	0.740
Monocyte count, ×10^3^/µL	600 (180–3020)	520 (220–1170)	0.061
Neutrophil count, ×10^3^/µL	4950 (411–17,670)	5070 (540–8500)	0.876
Platelet count, ×10^3^/µL	201.5 (71–519)	213 (69–398)	0.188
Glucose, mg/dL	112.5 (74–439)	111 (55–233)	0.673
Creatinine, mg/dL	1.00 (0.58–6.80)	1.02 (0.64–1.76)	0.669
Estimated GFR, mL/min/1.73 m^2^	56.15 ± 20.89	55.83 ± 16.77	0.938
Uric acid, mg/dL	7.48 ± 2.15	7.39 ± 1.68	0.832
Total protein, g/L	69 (52–101)	71.2 (60–79.3)	0.052
Albumin, g/L	38.09 ± 4.15	38.26 ± 4.04	0.843
C-reactive protein, mg/L	6 (0.1–185)	7 (0.2–80)	0.264
High-sensitivity troponin, ng/L	15.75 (0.8–16,118)	12 (2–2100)	0.864
Potassium, mmol/L	4.22 ± 0.55	4.31 ± 0.44	0.387
LDL cholesterol, mg/dL	105 (36–139)	96 (39–197)	0.051
HDL cholesterol, mg/dL	44.5 (22–87)	49 (31–76)	0.081
Triglycerides, mg/dL	128.5 (42–369)	101 (67–271)	0.092
Total cholesterol, mg/dL	168.5 (71–478)	157 (96–257)	0.225
Total bilirubin, mg/dL	0.59 (0.22–2.39)	0.72 (0.20–2.58)	0.226
**Echocardiographic parameters**			
Left ventricular ejection fraction, %	60 (20–65)	60 (30–65)	0.827
End-diastolic diameter, mm	47 (34–70)	46 (38–65)	0.496
End-systolic diameter, mm	30 (19–58)	30 (22–45)	0.750
Interventricular septum thickness, mm	14 (11–20)	14 (11–19)	0.902
Posterior wall thickness, mm	14 (11–18)	13 (10–19)	0.342
Left atrial diameter, mm	44.98 ± 6.19	45.87 ± 5.21	0.465
Pulmonary artery systolic pressure, mmHg	35 (15–85)	38 (18–65)	0.559
Left ventricular mass index, g/m^2^	147.1 (83–273)	141 (93–250)	0.083
Ascending aorta diameter, mm	37 (31–52)	36 (28–49)	0.272
Peak aortic jet velocity, cm/s	440 (310–590)	416 (331–730)	0.063
Peak aortic valve gradient, mmHg	74 (37–135)	69 (40–144)	0.170
Mean aortic valve gradient, mmHg	46 (21–89)	44 (30–93)	0.222
Aortic valve area, cm^2^	0.60 (0.30–1.00)	0.60 (0.30–0.90)	0.973
Carotid artery stenosis, n (%)	14 (12.1)	8 (25.8)	0.057
Logistic EuroSCORE II	14.89 ± 7.36	14.10 ± 7.14	0.594
STS score	3.10 (1.00–11.00)	3.42 (2.00–19.00)	0.149
Katz score			0.080
2, n (%)	2 (1.7)	0 (0.0)	
3, n (%)	3 (2.6)	1 (3.2)	
4, n (%)	4 (3.5)	4 (12.9)	
5, n (%)	6 (5.2)	5 (16.1)	
6, n (%)	99 (86.8)	21 (67.7)	
**Baseline electrocardiographic findings**			
Conduction disorder, n (%)	17 (14.6)	9 (29.0)	0.062
Left bundle branch block, n (%)	9 (7.7)	5 (16.1)	0.158
Right bundle branch block, n (%)	10 (8.6)	2 (6.4)	0.695
Atrial fibrillation, n (%)	46 (39.6)	11 (35.4)	0.765
Complete atrioventricular block, n (%)	1 (0.9)	1 (3.2)	0.299
Permanent pacemaker before TAVR, n (%)	4 (3.4)	1 (3.2)	0.952

Continuous variables are presented as mean ± standard deviation or median (range), according to their distribution. Categorical variables are presented as n (%). Continuous variables were compared using the Mann–Whitney *U* test, whereas categorical variables were compared using the chi-square test or Fisher’s exact test, as appropriate. Abbreviations: AF, atrial fibrillation; CRP, C-reactive protein; GFR, glomerular filtration rate; Hb, hemoglobin; HCT, hematocrit; HDL, high-density lipoprotein; hs-Troponin, high-sensitivity troponin; IVS, interventricular septum; LA, left atrium; LBBB, left bundle branch block; LDL, low-density lipoprotein; LVEF, left ventricular ejection fraction; LVMI, left ventricular mass index; PCI, percutaneous coronary intervention; PPM, permanent pacemaker; PWD, posterior wall diameter; RBBB, right bundle branch block; STS, Society of Thoracic Surgeons; TAVR, transcatheter aortic valve replacement; TG, triglycerides; WBC, white blood cell.

**Table 3:** Procedural Data.

Variable	Myval (n = 116)	Acurate Neo (n = 31)	*p*-Value
Procedure time, min	45 (20–80)	45 (30–140)	0.948
Contrast volume, mL	100 (25–240)	100 (40–155)	0.748
Predilatation, n (%)	22 (18.9)	31 (100.0)	**<0.001**
Postdilatation, n (%)	6 (5.2)	5 (16.1)	**0.039**
Preprocedural coronary angiography, n (%)	88 (75.8)	25 (80.6)	0.575
**Percutaneous vascular closure device**			**0.155**
Prostar, n (%)	6 (5.2)	0 (0.0)	0.196
ProGlide, n (%)	97 (83.6)	30 (96.7)	0.058
• Single ProGlide, n	45	0	
• Dual ProGlide, n	52	30	
Surgical vascular closure, n (%)	13 (11.2)	1 (3.2)	0.179

Continuous variables are presented as median (range). Categorical variables are presented as n (%). Continuous variables were compared using the Mann–Whitney *U* test, whereas categorical variables were compared using the chi-square test or Fisher’s exact test, as appropriate. Abbreviations: CAG, coronary angiography; mL, milliliter; min, minutes.

**Table 4:** Periprocedural outcomes according to the VARC-3 criteria.

Variable	Myval (n = 116)	Acurate Neo (n = 31)	*p*-Value
Periprocedural death, n (%)	5 (4.3)	2 (6.4)	0.619
Valve embolization, n (%)	1 (0.9)	0 (0.0)	0.604
Coronary artery obstruction, n (%)	1 (0.9)	0 (0.0)	0.604
Periprocedural myocardial infarction, n (%)	1 (0.9)	0 (0.0)	0.604
Suicide ventricle, n (%)	1 (0.9)	0 (0.0)	0.604
Cardiac tamponade, n (%)	4 (3.4)	0 (0.0)	0.295
Annular rupture, n (%)	0 (0.0)	0 (0.0)	—
Left ventricular perforation, n (%)	0 (0.0)	0 (0.0)	—
Ventricular arrhythmia, n (%)	0 (0.0)	0 (0.0)	—
**Final paravalvular leak**			**0.174**
└ None, n (%)	85 (72.6)	17 (54.8)	0.048
└ Mild, n (%)	24 (20.6)	12 (38.7)	0.038
└ Moderate or greater, n (%)	7 (6.0)	2 (6.4)	0.931
Technical success, n (%)	114 (98.2)	30 (96.7)	0.599

Categorical variables are presented as n (%). Comparisons between groups were performed using the chi-square test or Fisher’s exact test, as appropriate. Technical success and periprocedural outcomes were defined according to the Valve Academic Research Consortium-3 (VARC-3) criteria. Abbreviations: LV, left ventricle; MI, myocardial infarction; PVL, paravalvular leak; VARC-3, Valve Academic Research Consortium-3.

**Table 5:** Thirty-day clinical and echocardiographic outcomes according to the VARC-3 criteria.

Variable	Myval (n = 116)	Acurate Neo (n = 31)	*p* Value
Device success, n (%)	99 (85.3)	27 (87.1)	0.804
Early safety, n (%)	77 (66.4)	22 (70.9)	0.628
**Mortality**			**0.806**
Cardiac death, n (%)	6 (5.1)	2 (6.4)	0.780
Non-cardiac death, n (%)	3 (2.6)	0 (0.0)	0.366
**Neurological events**			**0.151**
Hemorrhagic stroke, n (%)	0 (0.0)	0 (0.0)	—
Ischemic stroke, n (%)	1 (0.9)	1 (3.2)	0.313
Transient ischemic attack, n (%)	1 (0.9)	1 (3.2)	0.313
Disabling stroke, n (%)	1 (0.9)	1 (3.2)	0.313
Non-disabling stroke, n (%)	0 (0.0)	1 (3.2)	0.052
**Bleeding and transfusion**			**0.819**
Type 1, n (%)	17 (14.6)	4 (12.9)	0.804
Type 2, n (%)	6 (5.1)	2 (6.4)	0.780
Type 3, n (%)	0 (0.0)	0 (0.0)	—
Type 4, n (%)	1 (0.9)	1 (3.2)	0.313
**Vascular complications**			**0.352**
Major, n (%)	1 (0.9)	1 (3.2)	0.313
Minor, n (%)	17 (14.6)	6 (19.3)	0.522
Access-related nonvascular complications, n (%)	0 (0.0)	0 (0.0)	—
Acute kidney injury, n (%)	8 (6.9)	2 (6.4)	0.930
Valve thrombosis, n (%)	0 (0.0)	0 (0.0)	—
Valvular endocarditis, n (%)	0 (0.0)	0 (0.0)	—
Moderate or greater PVL, n (%)	7 (6.0)	2 (6.4)	0.931
Aortic valve mean gradient, mmHg	9 (4–28)	9 (3–24)	0.562
Aortic valve peak gradient, mmHg	19 (10–42)	17 (8–39)	0.336
**Conduction disturbances**			**0.477**
Left bundle branch block, n (%)	13 (11.2)	7 (22.8)	0.101
Right bundle branch block, n (%)	13 (11.2)	3 (9.6)	0.808
Atrial fibrillation, n (%)	49 (42.2)	13 (41.9)	0.914
Ventricular arrhythmia, n (%)	0 (0.0)	0 (0.0)	—
Complete atrioventricular block, n (%)	15 (12.9)	3 (9.6)	0.663
Permanent pacemaker implantation, n (%)	16 (13.7)	4 (12.9)	0.898
Rehospitalization, n (%)	5 (4.3)	1 (3.2)	0.801

Continuous variables are presented as median (range). Categorical variables are presented as n (%). Continuous variables were compared using the Mann–Whitney *U* test, whereas categorical variables were compared using the chi-square test or Fisher’s exact test, as appropriate. Device success and early safety were defined according to the Valve Academic Research Consortium-3 (VARC-3) criteria. Abbreviations: AF, atrial fibrillation; AV, atrioventricular; LBBB, left bundle branch block; PPM, permanent pacemaker implantation; PVL, paravalvular leak; RBBB, right bundle branch block; VARC-3, Valve Academic Research Consortium-3.

**Table 6:** Mid-term clinical and echocardiographic outcomes during the mean 18-month follow-up according to the VARC-3 criteria.

Variable	Myval (n = 116)	Acurate Neo (n = 31)	*p*-Value
**Clinical efficacy, n (%)**	74 (63.8)	20 (64.5)	0.941
**Combined clinical events**			
**Mortality**			**0.135**
Cardiac death, n (%)	16 (13.8)	2 (6.4)	0.268
Non-cardiac death, n (%)	9 (7.7)	1 (3.2)	0.373
Cardiovascular rehospitalization, n (%)	17 (15.9)	5 (17.8)	0.802
**Neurological events**			**0.780**
Hemorrhagic stroke, n (%)	0 (0.0)	0 (0.0)	—
Ischemic stroke, n (%)	4 (3.4)	1 (3.2)	0.952
Transient ischemic attack, n (%)	2 (1.8)	1 (3.2)	0.599
Disabling stroke, n (%)	3 (2.5)	1 (3.2)	0.846
Non-disabling stroke, n (%)	1 (0.9)	1 (3.2)	0.313
Valve thrombosis, n (%)	0 (0.0)	0 (0.0)	—
Valvular endocarditis, n (%)	0 (0.0)	0 (0.0)	—
Mild paravalvular leak, n (%)	23 (19.8)	12 (38.7)	**0.028**
Moderate or greater paravalvular leak, n (%)	8 (6.9)	4 (12.9)	0.278
Aortic valve mean gradient, mmHg	9 (5–26)	9 (5–26)	0.954
Aortic valve peak gradient, mmHg	20 (12–45)	18.5 (9–40)	0.114
Permanent pacemaker implantation, n (%)	17 (14.6)	4 (12.9)	0.804

Continuous variables are presented as median (range). Categorical variables are presented as n (%). Continuous variables were compared using the Mann–Whitney *U* test, whereas categorical variables were compared using the chi-square test or Fisher’s exact test, as appropriate. Clinical efficacy was defined according to the Valve Academic Research Consortium-3 (VARC-3) criteria. Abbreviations: PPM, permanent pacemaker implantation; PVL, paravalvular leak; TIA, transient ischemic attack; VARC-3, Valve Academic Research Consortium-3.

## Data Availability

The original contributions presented in this study are included in the article. Further inquiries can be directed to the corresponding author.
